# Crystal structure of 4-{2-[4-(di­methyl­amino)­phen­yl]diazen-1-yl}-1-methyl­pyridinium iodide

**DOI:** 10.1107/S2056989015023646

**Published:** 2015-12-19

**Authors:** Katherine Chulvi, Ana Costero, Luis E. Ochando, Pablo Gaviña

**Affiliations:** a Universitat de València, Instituto de Reconocimiento Molecular y Desarrollo Tecnológico, Doctor Moliner 50,46100, Burjassot,Valencia, Spain

**Keywords:** crystal structure, [DAZOP^+^][I^−^], NLO, dye, π–π inter­action, C—H⋯ π inter­actions, I⋯π inter­action

## Abstract

The mol­ecular geometry of the ionic title compound, C_14_H_17_N_4_^+^·I^−^ or DAZOP^+^·I^−^, is essentially featureless. Regarding the crystal structure, in addition to the obvious cation–anion Coulombic inter­actions, the packing is mostly directed by non-covalent inter­actions involving both ring systems, as well as the iodide anion. It consists of cationic mol­ecules aligned along [101] and disposed in an anti­parallel fashion while linked into π-bonded dimeric entities by a stacking contact involving symmetry-related phenyl rings, with a centroid–centroid distance of 3.468 (3) Å and a slippage of 0.951 Å. The dimers are, in addition, sustained by a number of C—H⋯I and I⋯π (I⋯centroid = 3.876 Å) inter­actions involving the anion. Finally, inter­dimeric contacts are of the C—H⋯I and C—H⋯π types.

## Related literature   

For the synthesis of precursors, see: Li *et al.* (1995 ▸). For spectroscopic properties of the title compound, see: Gonbeau *et al.* (1999 ▸). For general infomation on non-linear optical materials, see: Coradin *et al.* (1997 ▸); Mestechkin (2001 ▸); Nunzi *et al.* (2008 ▸). For general infomation on new photonic materials, see: Yu *et al.* (2013 ▸). For related structures, see: Cristian *et al.* (2004 ▸); Evans *et al.* (2001 ▸); Xu *et al.* (2012 ▸).
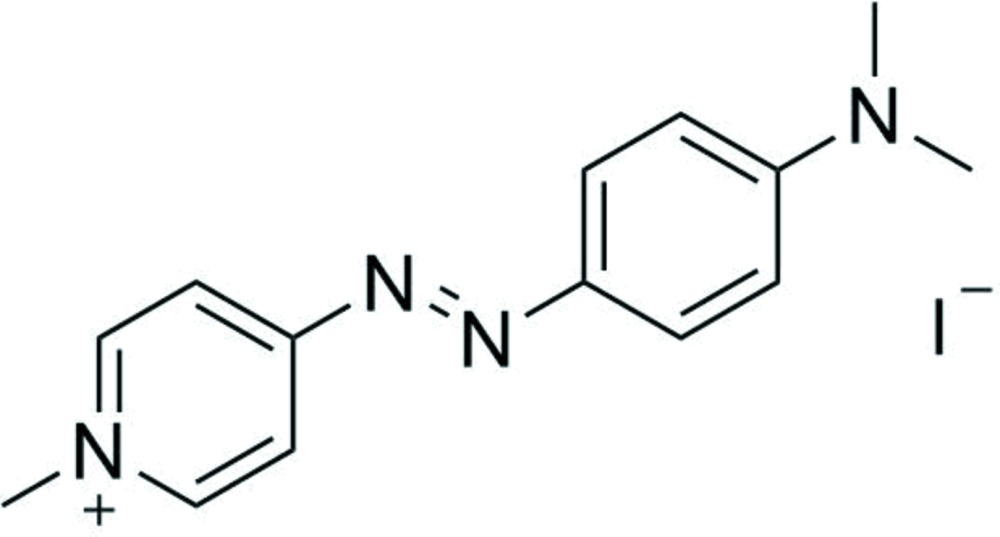


## Experimental   

### Crystal data  

C_14_H_17_N_4_^+^·I^−^
*M* *_r_* = 368.21
Monoclinic, 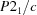

*a* = 18.0508 (14) Å
*b* = 7.2790 (5) Å
*c* = 11.3760 (9) Å
β = 98.929 (7)°
*V* = 1476.60 (19) Å^3^
*Z* = 4
Mo *K*α radiation
μ = 2.16 mm^−1^
*T* = 296 K
0.14 × 0.08 × 0.03 mm

### Data collection  

Agilent Xcalibur Sapphire3 Gemini diffractometer
Absorption correction: multi-scan (*CrysAlis PRO*; Agilent, 2009 ▸) *T*_min_ = 0.908, *T*_max_ = 1.000
5694 measured reflections
2591 independent reflections
1642 reflections with *I* > 2σ(*I*)
*R*_int_ = 0.048

### Refinement  

*R*[*F* ^2^ > 2σ(*F* ^2^)] = 0.035
*wR*(*F* ^2^) = 0.065
*S* = 0.78
2591 reflections
175 parameters
132 restraints
H-atom parameters constrained
Δρ_max_ = 1.00 e Å^−3^
Δρ_min_ = −0.51 e Å^−3^

### 

Data collection: *CrysAlis PRO* (Agilent, 2009 ▸); cell refinement: *CrysAlis PRO*); data reduction: *CrysAlis PRO*; program(s) used to solve structure: *SHELXS97* (Sheldrick, 2008 ▸); program(s) used to refine structure: *SHELXL2013* (Sheldrick, 2015 ▸); molecular graphics: *ORTEP-3 for Windows* (Farrugia, 2012 ▸); software used to prepare material for publication: *WinGX* (Farrugia, 2012 ▸).

## Supplementary Material

Crystal structure: contains datablock(s) I, shelx. DOI: 10.1107/S2056989015023646/bg2576sup1.cif

Structure factors: contains datablock(s) I. DOI: 10.1107/S2056989015023646/bg2576Isup2.hkl

**Fig. 1.**
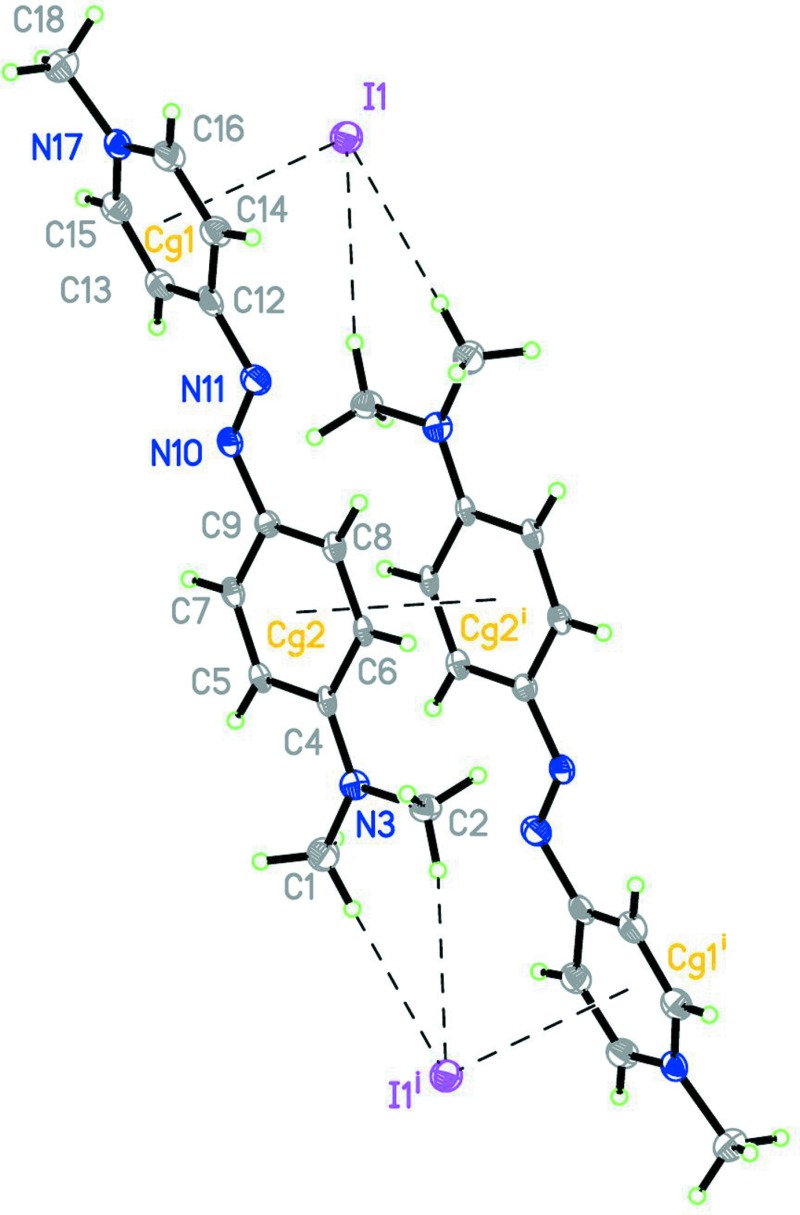
The molecular structure of (I),showing the atom-labelling scheme as well as the dimer formation. Displacement ellipsoids drawn at the 50% probability level. Symmetry codes: (i): 1 − x, 1 − y, 2 − z.

Supporting information file. DOI: 10.1107/S2056989015023646/bg2576Isup3.cml

x y z . DOI: 10.1107/S2056989015023646/bg2576fig1.tif
The mol­ecular structure of (I),showing the atom-labelling scheme as well as the dimer formation. Displacement ellipsoids drawn at the 50% probability level. Symmetry codes: (i): 1 − *x*, 1 − *y*, 2 − *z*.

CCDC reference: 1441443

Additional supporting information:  crystallographic information; 3D view; checkCIF report

## Figures and Tables

**Table 1:** Hydrogen-bond geometry (Å, °) *Cg*2 is the centroid of the C12–C16/N17 ring.

*D*—H⋯*A*	*D*—H	H⋯*A*	*D*⋯*A*	*D*—H⋯*A*
C1—H1*A*⋯I1^i^	0.96	3.09	4.042 (6)	173
C2—H2*A*⋯I1^i^	0.96	3.15	4.102 (5)	169
C15—H15*A*⋯I1^ii^	0.93	2.99	3.907 (5)	171
C7—H7*A*⋯*Cg*2^iii^	0.93	2.71	3.505 (5)	143

Symmetry codes: (i) 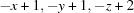; (ii) 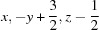; (iii) 
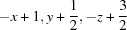
.

## supplementary crystallographic information

### S1. Chemical context

Over the years, the spectroscopic properties of 4-[2-(4-di­methyl­amino­phenyl)­azo]-1-methyl­pyridinium iodide ([DAZOP^+^][I^−^]) have been widely studied (Gonbeau *et al.*, 1999). This dye with donor-acceptor character, belongs to the group of the so-called non-linear optical chromophores (NLO-phore) that are able to form J-type aggregates (Coradin *et al.*, 1997; Mestechkin, 2001; Nunzi *et al.*, 2008). The crystal structures of this kind of NLO dyes are a topic of inter­est in this context and also for studies related to the solvatochromic properties of these dyes in hydrogen-bond-donor (HBD) and hydrogen-bond-acceptor (HBA) solvents. Very recently, new three-dimensional materials based on organic metalorganic frameworks (MOFs) with the capability to encapsulate dyes have been developed. All of these progress are aimed to the new photonic materials and devices design (Yu *et al.*, 2013).

### S2. Structural commentary

The title ionic compound [DAZOP^+^][I^−^] (I) crystallizes in the monoclinic S·G. P21/c, and presents one single molecule in the asymmetric unit. The molecular geometry, presented in Fig. 1, is essentially featureless.

### S3. Supra­molecular features

In addition to the obvious cation-anion coulombian inter­actions, the crystal packing is mostly directed by non covalent inter­actions involving the ring systems *Cg*1 (C4->C9) and *Cg*2 (C12->C16,N17, as well as the Iodine anion.

It consists of cationic molecules aligned along the [101] direction and disposed in an anti­parallel fashion while linked into π bonded dimeric entities (Fig. 1) by a stacking contact involving *Cg*2 and *Cg*2^i^ [(i): 1 − *x*,1 − *y*,2 − *z*)]), with d(*Cg*···*Cg*) = 3.468 (3) Å and a slippage of 0.951 Å. The dimer is in addition sustained by a number of inter­actions involving I1, *viz* (*a*) I···*Cg*1^i^, with d(I···*Cg*)= 3.876 Å, (*b*) C1—H1A···I1^i^, with d(H···I) = 3.09 Å, <C—H···I> = 173°, (*c*) C2—H2A···I1^i^, with d(H···I) = 3.15 Å, <C—H···I> = 169°. The remaining non-covalent inter­actions serve to link these dimers with each other, either directly, *viz*., through a C7—H7···*Cg*2iii [(iii): 1 − *x*,1/2 + *y*,3/2 − *z*] contact, with d(H···*Cg*) = 2.71 Å, <C—H···*Cg*> = 143° or mediated by the external iodine (*viz*., C15—H15.. I1^ii^ [(ii): *x*,3/2 − *y*,-1/2 + *z*], d(H···I): 2.99 Å; <C—H···I> = 171°).

### S4. Database survey

There are in the literature a lot of crystal structures derived from DAZOP but none with iodine as counter ion. The most similar to (I) is the one with CSD code (Allen, 2002) HANKUD (Cristian *et al.*, 2004) which was solved using powder data and contains a molecule of hexa­fluoro­phosphate as counter ion. Thus although the molecules are practically the same, the differences between both structures are significant, mainly due to the absence of π–π inter­actions in HANKUD. Some similar structures of (DAZOP^+^) coordinated with metalorganic ions can be found in the CSD, *viz*., IFAHAY (J. S. O. Evans *et al.*, 2001), RARTEL, RARTIP, RARTOV, (Xu *et al.*, 2012), *etc*.

### S5. Synthesis and crystallization

Benzenamine, *N*,*N*-di­methyl-4- (4-pyridinylazo)- was obtained as described in the literature (Li *et al.*, 1995). It was then dissolved in aceto­nitrile and stirred while an excess of methyl iodide was added dropwise. The resultant mixture was refluxed for 3 h. After that, the orange precipitated obtained was further purified by column chromatography (1:4, methanol/ethyl acetate) with a yield of 59%. Single crystals were obtained by slow evaporation from a methanol solution using a Petri dish.

### S6. Refinement

Crystal data, data collection and structure refinement details are summarized in Table 1

### Crystal data

**Table:**

C_14_H_17_N_4_^+^·I^−^	*F*(000) = 728
*M**_r_* = 368.21	*D*_x_ = 1.656 Mg m^−^^3^
Monoclinic, *P*2_1_/*c*	Mo *K*α radiation, λ = 0.71073 Å
*a* = 18.0508 (14) Å	Cell parameters from 2009 reflections
*b* = 7.2790 (5) Å	θ = 2.3–29.8°
*c* = 11.3760 (9) Å	µ = 2.16 mm^−^^1^
β = 98.929 (7)°	*T* = 296 K
*V* = 1476.60 (19) Å^3^	Plate, orange
*Z* = 4	0.14 × 0.08 × 0.03 mm

### Data collection

**Table:**

Agilent Xcalibur Sapphire3 Gemini diffractometer	2591 independent reflections
Radiation source: Enhance (Mo) X-ray Source	1642 reflections with *I* > 2σ(*I*)
Detector resolution: 16.0267 pixels mm^-1^	*R*_int_ = 0.048
ω scans	θ_max_ = 25.0°, θ_min_ = 2.3°
Absorption correction: multi-scan (*CrysAlis PRO*; Agilent, 2009)	*h* = −21→17
*T*_min_ = 0.908, *T*_max_ = 1.000	*k* = −6→8
5694 measured reflections	*l* = −13→10

### Refinement

**Table:**

Refinement on *F*^2^	132 restraints
Least-squares matrix: full	Hydrogen site location: inferred from neighbouring sites
*R*[*F*^2^ > 2σ(*F*^2^)] = 0.035	H-atom parameters constrained
*wR*(*F*^2^) = 0.065	*w* = 1/[σ^2^(*F*_o_^2^) + (0.0256*P*)^2^] where *P* = (*F*_o_^2^ + 2*F*_c_^2^)/3
*S* = 0.78	(Δ/σ)_max_ = 0.001
2591 reflections	Δρ_max_ = 1.00 e Å^−^^3^
175 parameters	Δρ_min_ = −0.51 e Å^−^^3^

### Special details

**Table:**

Geometry. All e.s.d.'s (except the e.s.d. in the dihedral angle between two l.s. planes) are estimated using the full covariance matrix. The cell e.s.d.'s are taken into account individually in the estimation of e.s.d.'s in distances, angles and torsion angles; correlations between e.s.d.'s in cell parameters are only used when they are defined by crystal symmetry. An approximate (isotropic) treatment of cell e.s.d.'s is used for estimating e.s.d.'s involving l.s. planes.

### Fractional atomic coordinates and isotropic or equivalent isotropic displacement parameters (Å^2^)

**Table:**

	*x*	*y*	*z*	*U*_iso_*/*U*_eq_	
C1	0.7113 (3)	0.4360 (7)	0.9607 (5)	0.0253 (13)	
H1A	0.7574	0.4185	1.0140	0.038*	
H1B	0.6980	0.5639	0.9585	0.038*	
H1C	0.7173	0.3960	0.8824	0.038*	
C2	0.6716 (3)	0.2294 (6)	1.1152 (5)	0.0200 (13)	
H2A	0.7244	0.2417	1.1432	0.030*	
H2B	0.6593	0.1018	1.1030	0.030*	
H2C	0.6439	0.2796	1.1732	0.030*	
N3	0.6522 (2)	0.3295 (6)	1.0020 (4)	0.0184 (9)	
C4	0.5810 (3)	0.3369 (6)	0.9457 (4)	0.0129 (9)	
C5	0.5610 (3)	0.4377 (6)	0.8383 (4)	0.0152 (9)	
H5A	0.5978	0.5008	0.8058	0.018*	
C6	0.5211 (3)	0.2442 (6)	0.9920 (5)	0.0141 (10)	
H6A	0.5316	0.1782	1.0627	0.017*	
C7	0.4886 (3)	0.4430 (6)	0.7824 (4)	0.0155 (10)	
H7A	0.4773	0.5105	0.7125	0.019*	
C8	0.4502 (3)	0.2522 (6)	0.9340 (5)	0.0152 (10)	
H8A	0.4127	0.1908	0.9660	0.018*	
C9	0.4305 (3)	0.3502 (6)	0.8265 (4)	0.0131 (9)	
N10	0.3607 (2)	0.3636 (5)	0.7569 (4)	0.0180 (9)	
N11	0.3082 (2)	0.2693 (5)	0.7907 (4)	0.0215 (9)	
C12	0.2413 (3)	0.2902 (7)	0.7100 (4)	0.0198 (10)	
C13	0.2268 (3)	0.4246 (7)	0.6216 (5)	0.0219 (11)	
H13A	0.2625	0.5145	0.6150	0.026*	
C14	0.1827 (3)	0.1655 (7)	0.7185 (5)	0.0220 (11)	
H14A	0.1880	0.0799	0.7799	0.026*	
C15	0.1614 (3)	0.4251 (7)	0.5455 (5)	0.0211 (11)	
H15A	0.1527	0.5167	0.4881	0.025*	
C16	0.1185 (3)	0.1683 (7)	0.6385 (5)	0.0226 (10)	
H16A	0.0813	0.0819	0.6441	0.027*	
N17	0.1081 (2)	0.2955 (6)	0.5508 (4)	0.0184 (8)	
C18	0.0410 (3)	0.2872 (7)	0.4591 (5)	0.0260 (13)	
H18A	0.0347	0.4024	0.4178	0.039*	
H18B	−0.0023	0.2629	0.4961	0.039*	
H18C	0.0469	0.1908	0.4037	0.039*	
I1	0.10101 (2)	0.68987 (5)	0.81084 (3)	0.02305 (12)	

### Atomic displacement parameters (Å^2^)

**Table:**

	*U*^11^	*U*^22^	*U*^33^	*U*^12^	*U*^13^	*U*^23^
C1	0.0259 (19)	0.028 (3)	0.022 (3)	−0.0084 (19)	0.004 (2)	0.003 (2)
C2	0.018 (3)	0.021 (3)	0.0204 (17)	−0.001 (2)	0.0039 (14)	0.0044 (16)
N3	0.0204 (12)	0.016 (2)	0.0187 (16)	−0.0011 (11)	0.0036 (10)	0.0014 (15)
C4	0.0196 (12)	0.009 (2)	0.0116 (15)	0.0006 (11)	0.0060 (10)	−0.0040 (14)
C5	0.0206 (11)	0.013 (2)	0.0132 (15)	0.0011 (13)	0.0063 (11)	−0.0014 (15)
C6	0.0208 (13)	0.010 (2)	0.0125 (18)	−0.0006 (11)	0.0069 (10)	−0.0044 (15)
C7	0.0208 (11)	0.012 (2)	0.015 (2)	0.0010 (12)	0.0056 (10)	−0.0012 (17)
C8	0.0212 (13)	0.009 (2)	0.0159 (15)	−0.0013 (13)	0.0056 (12)	−0.0028 (14)
C9	0.0213 (11)	0.005 (2)	0.0140 (15)	0.0020 (11)	0.0076 (11)	−0.0056 (13)
N10	0.0219 (11)	0.017 (2)	0.0161 (17)	0.0021 (11)	0.0066 (10)	−0.0039 (14)
N11	0.0231 (11)	0.022 (2)	0.0211 (18)	0.0007 (11)	0.0073 (11)	−0.0010 (14)
C12	0.0221 (12)	0.0215 (19)	0.0178 (17)	0.0026 (12)	0.0092 (12)	−0.0038 (15)
C13	0.0215 (18)	0.024 (2)	0.0211 (19)	0.0008 (14)	0.0074 (14)	−0.0008 (16)
C14	0.0211 (13)	0.023 (2)	0.023 (2)	0.0027 (14)	0.0053 (13)	0.0022 (16)
C15	0.0223 (16)	0.019 (2)	0.023 (2)	0.0016 (13)	0.0062 (13)	−0.0021 (16)
C16	0.0214 (16)	0.024 (2)	0.0232 (18)	0.0021 (14)	0.0048 (14)	0.0031 (16)
N17	0.0208 (15)	0.0174 (17)	0.0178 (17)	0.0030 (13)	0.0056 (12)	−0.0027 (13)
C18	0.0255 (18)	0.027 (3)	0.024 (2)	−0.0005 (19)	0.0002 (16)	0.001 (2)
I1	0.0222 (2)	0.0228 (2)	0.0241 (2)	−0.0007 (2)	0.00320 (14)	−0.0028 (2)

### Geometric parameters (Å, º)

**Table:**

C1—N3	1.454 (6)	C8—H8A	0.9300
C1—H1A	0.9600	C9—N10	1.383 (6)
C1—H1B	0.9600	N10—N11	1.277 (5)
C1—H1C	0.9600	N11—C12	1.408 (6)
C2—N3	1.474 (6)	C12—C13	1.398 (7)
C2—H2A	0.9600	C12—C14	1.408 (7)
C2—H2B	0.9600	C13—C15	1.351 (7)
C2—H2C	0.9600	C13—H13A	0.9300
N3—C4	1.344 (6)	C14—C16	1.358 (7)
C4—C5	1.423 (7)	C14—H14A	0.9300
C4—C6	1.442 (6)	C15—N17	1.355 (6)
C5—C7	1.361 (6)	C15—H15A	0.9300
C5—H5A	0.9300	C16—N17	1.352 (6)
C6—C8	1.348 (7)	C16—H16A	0.9300
C6—H6A	0.9300	N17—C18	1.471 (6)
C7—C9	1.405 (6)	C18—H18A	0.9600
C7—H7A	0.9300	C18—H18B	0.9600
C8—C9	1.413 (7)	C18—H18C	0.9600
			
N3—C1—H1A	109.5	N10—C9—C7	115.2 (4)
N3—C1—H1B	109.5	N10—C9—C8	128.0 (5)
H1A—C1—H1B	109.5	C7—C9—C8	116.8 (5)
N3—C1—H1C	109.5	N11—N10—C9	116.2 (4)
H1A—C1—H1C	109.5	N10—N11—C12	110.4 (4)
H1B—C1—H1C	109.5	C13—C12—N11	126.2 (5)
N3—C2—H2A	109.5	C13—C12—C14	116.2 (5)
N3—C2—H2B	109.5	N11—C12—C14	117.6 (5)
H2A—C2—H2B	109.5	C15—C13—C12	120.6 (5)
N3—C2—H2C	109.5	C15—C13—H13A	119.7
H2A—C2—H2C	109.5	C12—C13—H13A	119.7
H2B—C2—H2C	109.5	C16—C14—C12	121.1 (5)
C4—N3—C1	121.2 (4)	C16—C14—H14A	119.5
C4—N3—C2	121.1 (4)	C12—C14—H14A	119.5
C1—N3—C2	117.3 (4)	C13—C15—N17	121.7 (5)
N3—C4—C5	121.8 (4)	C13—C15—H15A	119.1
N3—C4—C6	121.5 (4)	N17—C15—H15A	119.1
C5—C4—C6	116.7 (5)	N17—C16—C14	120.7 (5)
C7—C5—C4	120.9 (5)	N17—C16—H16A	119.6
C7—C5—H5A	119.6	C14—C16—H16A	119.6
C4—C5—H5A	119.6	C16—N17—C15	119.4 (5)
C8—C6—C4	120.7 (5)	C16—N17—C18	120.0 (4)
C8—C6—H6A	119.7	C15—N17—C18	120.6 (4)
C4—C6—H6A	119.7	N17—C18—H18A	109.5
C5—C7—C9	122.4 (5)	N17—C18—H18B	109.5
C5—C7—H7A	118.8	H18A—C18—H18B	109.5
C9—C7—H7A	118.8	N17—C18—H18C	109.5
C6—C8—C9	122.5 (5)	H18A—C18—H18C	109.5
C6—C8—H8A	118.7	H18B—C18—H18C	109.5
C9—C8—H8A	118.7		
			
C1—N3—C4—C5	4.8 (7)	C8—C9—N10—N11	−3.6 (7)
C2—N3—C4—C5	178.0 (4)	C9—N10—N11—C12	−177.8 (4)
C1—N3—C4—C6	−174.6 (4)	N10—N11—C12—C13	−14.3 (7)
C2—N3—C4—C6	−1.4 (7)	N10—N11—C12—C14	166.1 (4)
N3—C4—C5—C7	179.8 (4)	N11—C12—C13—C15	176.8 (5)
C6—C4—C5—C7	−0.8 (7)	C14—C12—C13—C15	−3.6 (7)
N3—C4—C6—C8	−179.6 (4)	C13—C12—C14—C16	5.2 (7)
C5—C4—C6—C8	1.0 (7)	N11—C12—C14—C16	−175.2 (5)
C4—C5—C7—C9	−0.3 (7)	C12—C13—C15—N17	−0.8 (8)
C4—C6—C8—C9	−0.1 (7)	C12—C14—C16—N17	−2.3 (8)
C5—C7—C9—N10	−177.6 (4)	C14—C16—N17—C15	−2.2 (7)
C5—C7—C9—C8	1.1 (7)	C14—C16—N17—C18	174.9 (5)
C6—C8—C9—N10	177.6 (4)	C13—C15—N17—C16	3.9 (7)
C6—C8—C9—C7	−0.9 (7)	C13—C15—N17—C18	−173.3 (5)
C7—C9—N10—N11	175.0 (4)		

### Hydrogen-bond geometry (Å, º)

Cg2 is the centroid of the C12–C16/N17 ring.

**Table:**

*D*—H···*A*	*D*—H	H···*A*	*D*···*A*	*D*—H···*A*
C1—H1*A*···I1^i^	0.96	3.09	4.042 (6)	173
C2—H2*A*···I1^i^	0.96	3.15	4.102 (5)	169
C15—H15*A*···I1^ii^	0.93	2.99	3.907 (5)	171
C7—H7*A*···*Cg*2^iii^	0.93	2.71	3.505 (5)	143

Symmetry codes: (i) −*x*+1, −*y*+1, −*z*+2; (ii) *x*, −*y*+3/2, *z*−1/2; (iii) −*x*+1, *y*+1/2, −*z*+3/2.
