# Supplementary figures and images for: Crystal structure of 4-{2-[4-(di­methyl­amino)­phen­yl]diazen-1-yl}-1-methyl­pyridinium iodide
Source: Acta Crystallogr E Crystallogr Commun. 2015 Dec 19;71(Pt 12):o1069–70. doi: 10.1107/S2056989015023646 (PMC4719983; doi:10.1107/S2056989015023646)

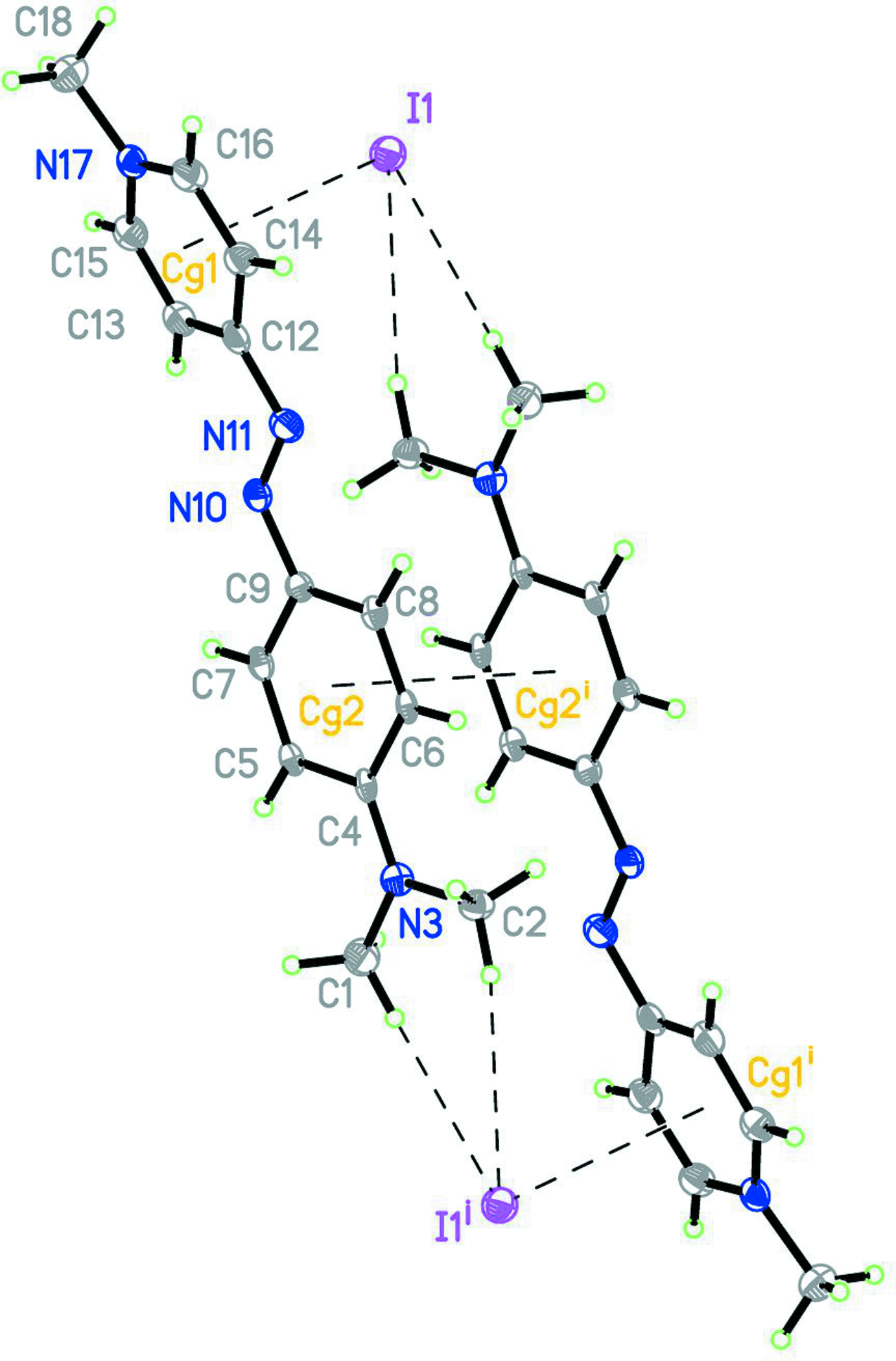

Supplement: Supplementary file 4 [file e-71-o1069-fig1.tif]
